# Assessment of Chemopreventive Potential of the Plant Extracts against Liver Cancer Using HepG2 Cell Line

**DOI:** 10.3390/molecules26154593

**Published:** 2021-07-29

**Authors:** Deepthi Venkatachalapathy, Chandan Shivamallu, Shashanka K. Prasad, Gopenath Thangaraj Saradha, Parthiban Rudrapathy, Raghavendra G. Amachawadi, Sharanagouda S. Patil, Asad Syed, Abdallah M. Elgorban, Ali H. Bahkali, Shiva Prasad Kollur, Kanthesh M. Basalingappa

**Affiliations:** 1 Division of Molecular Biology, Faculty of Life Science, JSS Academy of Higher Education & Research, Mysuru 570015, India; deepthivdarsh@gmail.com; 2 Department of Biotechnology and Bioinformatics, School of Life Sciences, JSS Academy of Higher Education and Research, Mysuru 570105, India; shashankaprasad@jssuni.edu.in (S.K.P.); gopenath@jssuni.edu.in (G.T.S.); 3 Malabar Cancer Centre, Department of Clinical Laboratory Services & Translational Research, Thalassery, Kannur 670103, India; parthi71975@gmail.com; 4 Department of Clinical Sciences, College of Veterinary Medicine, Kansas State University, Manhattan, KS 66506-5606, USA; agraghav@vet.k-state.edu; 5 ICAR, National Institute of Veterinary Epidemiology and Disease Informatics (NIVEDI), Yelahanka, Bengaluru 560064, India; sharanspin13@gmail.com; 6 Department of Botany and Microbiology, College of Science, King Saud University, P.O. Box 2455, Riyadh 11451, Saudi Arabia; assyed@ksu.edu.sa (A.S.); aelgorban@ksu.edu.sa (A.M.E.); abahakli@ksu.edu.sa (A.H.B.); 7 Department of Sciences, Amrita School of Arts and Sciences, Amrita Vishwa Vidyapeetham, Mysuru Campus, Mysuru 570026, India

**Keywords:** *Camellia sinensis*, *Withania somnifera*, *Vitis vinifera*, anti-carcinogenic, antioxidants, HepG2 cell line

## Abstract

The edible parts of the plants *Camellia sinensis*, *Vitis vinifera* and *Withania somnifera* were extensively used in ancient practices such as Ayurveda, owing to their potent biomedical significance. They are very rich in secondary metabolites such as polyphenols, which are very good antioxidants and exhibit anti-carcinogenic properties. This study aims to evaluate the anti-cancerous properties of these plant crude extracts on human liver cancer HepG2 cells. The leaves of *Camellia sinensis*, *Withania somnifera* and the seeds of *Vitis vinifera* were collected and methanolic extracts were prepared. Then, these extracts were subjected to DPPH, α- amylase assays to determine the antioxidant properties. A MTT assay was performed to investigate the viability of the extracts of HepG2 cells, and the mode of cell death was detected by Ao/EtBr staining and flow cytometry with PI Annexin- V FITC dual staining. Then, the protein expression of BAX and BCl2 was studied using fluorescent dye to determine the regulation of the BAX and BCl2 genes. We observed that all the three extracts showed the presence of bioactive compounds such as polyphenols or phytochemicals. The *W. somnifera* bioactive compounds were found to have the highest anti-proliferative activity on human liver cancer cells.

## 1. Introduction

The disease most commonly causing death is cancer. Cancer is most commonly being witnessed in developed countries, with liver cancer being the third most common cause of death worldwide. However, there are standard protocols for the treatment of cancer, such as chemotherapy or radiotherapy, they are accompanied by severe physical and physiological side effects. With this background of treating cancer, recent advancements have been developed which include the isolation and identification of phytochemical compounds for treating cancer [1,2]. This has become a recent trend in the last two decades in the field of the potential use and the application of traditional and herbal plants such as *Andrographis paniculata*, *Centella asiatica,* etc. as anticancer agents [3]. Several medicinal plants have been found to possess anticancer properties. Different parts of plants containing various bioactive compounds exhibit significant inhibition of cancer [4]. The use of natural compounds for the chemoprevention of cancer is a promising strategy against the initiation of cancer [5]. There is evidence accumulating over the increase in the use of the medicinal plant worldwide and serving as the source to produce drugs [6]. Though there are several approaches to drug discovery, such as combinatorial chemistry and computer-based molecule modelling design [7], the characteristic features and the structural diversity of the bioactive compounds present in plants make them a useful source for the novel therapeutics.

The naturally occurring antioxidants in food and nutraceuticals are said to replace synthetic antioxidants since they are safer and healthier, without any adverse effects, unlike synthetic antioxidants. These antioxidants are the biomolecules that serve as scavengers of the free radicals being produced by the body’s cells during several metabolic processes and due to by several external factors. These free radicals, being unstable, are destructive for the proteins, lipids, DNA and RNA present in the human body. These free radicals are one of the main causes of the initiation of cancer in the human body [8]. These antioxidants play a major role in preventing the onset of carcinogenesis and are thus beneficial to the cells. *Camellia sinensis* is an evergreen, slow-growing, dwarf shrub. It is native to China and South East Asia [9]. *Camellia sinensis* belongs to the family Theaceae, with most of the species coming from China, Sri Lanka, Japan, and India [10]. It is a shrub commonly known as the grapevine: it is one of the most economically important fruit crops, having about 60 different species in the genus *Vitis* [11,12]. *Vitis vinifera* is a fast rate deciduous climber, growing up to 15 m. *Withania somnifera* is commonly known as the “Indian Cherry’’ which is the most important Indian traditional herb in Ayurvedic medicine [13]. It is a medical plant which usually grows on dry land in the northwestern regions of the Punjab and Jammu. It is cultivated at a high altitude of 1500 m. It is usually grown in the frequently drought-stricken central and southern states of India [14]. In the current research paper, we aim to evaluate the anti-cancerous properties of these plant crude extracts on human liver cancer HepG2 cells.

## 2. Materials and Methods

### 2.1. Sample Collection

We evaluated fresh leaves of *Camellia sinensis* grown in Mara in the state of Mizoram, India. They were verified and received by the organic certified D and G Biotech company, which is FSSAI approved. We evaluated fresh leaves of *Withania somnifera* grown in the region of Neemuch town in the state of Madhya Pradesh. They were verified and received from the registered herbal trading company called in February 2020. The dry seeds of *Vitis vinifera* were collected from Grover Zampa vineyard, a wine-producing company.

### 2.2. Sample Preparation and Extraction

**Figure 1:**
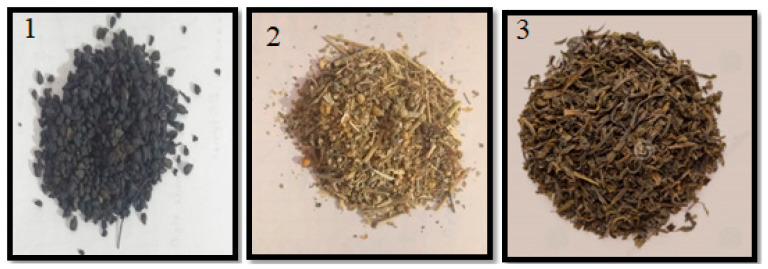
Dry leaves of 1. *Vitis vinifera*, 2. *Withania somnifera*, 3. *Camelia sinensis*.

All the leaves (Figure 1) were washed with tap water and dried at normal room temperature for a week. The dried plant samples were crushed and powdered manually and with the use of a mixer for a few seconds to turn the seeds into powder. The powdered plant samples were filtered using a sieve to remove larger lumps. Finally, the finely powdered samples were collected for further processing. We weighed 20 g of dried sample powder and dissolved it in 100 mL of methanol in a 500 mL beaker covered with aluminium foil. Then, the beaker was kept in a hot water bath at 50 °C for 4 h. After the incubation period, the extract was filtered with Whatmann filter paper No. 1 and the filtrate was collected in a 50 mL beaker. Residue present on the filter paper was discarded and the filtrate was taken for further use. Then, the filtrate was kept at 50 °C for a few hours until the extract was completely dried and turned into a semisolid form. The obtained semisolid state is the plant extract which was weighed and the yield was noted and stored at room temperature until the next use. 

### 2.3. Phytochemical Screening of Extracts and Antioxidant Activity Using DPPH Assay 

Phytochemical screening was performed using standard procedures. Samples of the methanolic solvent were screened for the following phytoconstituents: alkaloids, carbohydrates, tannins, saponins, terpenoids, flavonoids, tannins, cardiac glycosides.

Test for Alkaloids: Dragendoff’s test: We took 0.2 mL of sample and added 0.2 mL of HCl. To these 2–3 drops of Dragendoff’s reagent was added and the appearance of orange or red precipitate and turbid solution indicates the presence of alkaloids.

Test for Carbohydrates: Molisch’s test: We mixed 0.2 mL of sample with a few drops of Molisch’s reagent (α-naphthol dissolved in alcohol). We added 0.2 mL of sulphuric acid along the sides of the test tube and observed it for the appearance of a purple colour ring indicating a positive test.

Test for Terpenoids: Salkowki’s test: We placed 0.2 mL of plant extract in a test tube with 0.2 mL of chloroform. To this, concentrated sulphuric acid was added carefully to form a layer. Presence of reddish brown colour at the interface would show the presence of terpenoids.

Test for Glycosides: We mixed 0.2 mL of sample with 0.2 mL of chloroform. We added 0.2 mL of acetic acid to this solution and the mixture was cooled on ice. Sulphuric acid was added carefully and the colour change from violet to blue to green indicates the presence of steroidal nucleus (a glycone portion of glycoside).

Test for Steroids: Lieberman Burchardt tests: We mixed 0.2 mL of sample with 0.2 mL of chloroform. To this, 0.2 mL of concentrated sulphuric acid was added. The appearance of red colour in the lower layer of chloroform indicates the presence of steroids.

Test for Saponins: Test for Saponins (Foam test): To 0.2 mL of extract was added 0.6 mL of water in a test tube. The mixture was shaken vigorously and observed for the formation of persistent foam that confirms the presence of saponins.

Test for Flavonoids: Alkaline reagent test: We placed 0.2 mL of plant extract in a test tube and mixed with dilute sodium hydroxide solution. To this diluted mixture, hydrochloric acid was added. Observation of yellow solution that turn colourless later would indicate the presence of flavonoids.

Mucilage test (Glycoprotein): We placed 0.2 mL of extract in a test tube and 0.2 mL of absolute alcohol was added and allowed to dry. If the precipitation occurs then mucilage is present.

Volatile oil: We treated 0.2 mL of extract with a few drops of dilute hydrochloric acid. The appearance of white precipitate indicates the presence of volatile oils.

Test for phenols: To 0.2 mL of extract 0.4 mL of distilled water followed by a few drops of 10% aqueous ferric chloride solution were added. Formation of blue or green colour indicates the presence of phenols.

DPPH assay is carried out as per the method of Rajakumar et al. [15]. DPPH (EEC No. 217-591-8, Sigma, St. Louis, MO, USA), stored at less than 0 °C, methanol, HPLC grade (Ranbaxy Chemicals) was used for the DPPH analysis. Quercetin (1 mg/mL) was taken as the reference standard for use for the DPPH assay. DPPH1 mg/dissolved in 6 mL HPLC grade methanol and Quercetin1 mg dissolved in 1 mL methanol was prepared as working solutions.

In brief, 80 μL of DPPH solution; various concentrations of test solution and quantities sufficient for 240 μL with HPLC grade methanol. The different concentrations tested for reference standard were 0.3125, 0.625, 1.25, 2.5, 5, 10 µg/mL. The reaction mixture was mixed and incubated at 25 °C for 15 min. The absorbance was measured at 510 nm using a Spectramax I3x multimode microplate reader from Bio-Rad (102). A control reaction was carried out without the test sample.

IC (50) value was calculated using Graph Prism software version 5.0 by nonlinear regression analysis of % inhibition recorded for different concentrations of test substances/standard. For compounds showing ‹50% inhibition, the IC50 value was not calculated. The relative activity of the sample can be determined by comparing the IC50 value of the sample with a standard. The higher the IC50 value, the lower will be the relative activity in comparison to standard and amp, and vice versa.

A two-way ANOVA between the different concentrations and samples was conducted to compare the effect of samples on DPPH scavenging activity and significant effect of different samples was observed at *p* < 0.05. 

#### 2.3.1. α-. Amylase Assay

The pre-incubated 120 µL phosphate buffer with pH 7.0 along with test sample/reference standard of various concentrations and 60 µL of the enzyme was left at 37 °C for 10 min. Then, 250 µL of substrate reagent (CNPG3) was added and incubated at 37 °C for 8 min. The reaction was arrested by heating in a boiling water bath for 2 min and cooled. The absorbance was measured at 405 nm. A control reaction was carried out without the test sample. 

#### 2.3.2. HPLC Analysis 

The powdered test samples, each 10 g, were extracted with methanol and incubated at 50 °C and filtered using Whatman filter paper. The filtrates were kept for evaporation in a boiling water bath of 70 °C. Extracts of 10 µL were injected into HPLC. The instrument of HPLC used was Shimadzhu LC- prominence 20AT with a C18 column of 250 mm × 4.6 mm, 5µ particle size in the stationary phase. In linear mobile phase, 50% of acetonitrile and 50% of HPLC grade water was used. The flow rate was 1 mL/min with an absorbance at 254 nm.

#### 2.3.3. MTT Assay for HepG2 Cell Line 

We took 10 mM stock of doxorubicin. Further Serial two-fold dilutions were prepared from 100 µM to 3.125 µM using Dulbecco’s Modified Eagle Medium (DMEM) for treatment. They were 100, 50, 25, 12.5, 6.25 and 3.125 µM. For cytotoxicity studies, 32 mg/mL stocks were prepared using DMSO. Serial two-fold dilutions were prepared from 320 µg/mL to 10 µg/mL using DMEM plain media for treatment. They were 32 mg/mL, 3.2 mg/mL, 320 mg/mL, 160 mg/mL, 80 mg/mL, 40 mg/mL, 20 mg/mL, and 10 mg/mL.

#### 2.3.4. Preparation of Cell Lines and Culture Medium for MTT Assay

HepG-2 cell lines were procured from ATCC, stock cells were cultured in DMEM supplemented with 10% inactivated Foetal Bovine Serum (FBS), penicillin (100 IU/mL), streptomycin (100 µg/mL) in a humidified atmosphere of 5% CO_2_ at 37 °C until confluent. The cell was dissociated with cell dissociating solution (0.2% trypsin, 0.02% EDTA, 0.05% glucose in PBS). The viability of the cells was checked before they were centrifuged. Further, 50,000 cells/well were seeded in a 96-well plate and incubated for 24 h at 37 °C, 5% CO_2_ incubator. The IC_50_ values for cytotoxicity tests were derived from nonlinear regression analysis (curve fit) based on the sigmoid dose–response curve (variable) and computed using Graph Pad Prism 6 (Graph pad, San Diego, CA, USA) [16,17,18,19,20,21,22].

#### 2.3.5. Acridine Orange and Ethidium Bromide Dual Staining Studies Using HepG2 Cell Line

First, 25 µL (approx. 1 × 10^5^ cells) of treated and untreated cells were taken separately in a microcentrifuge tube and stained with 5 µL of AO-EtBr (acridine orange and ethidium bromide) for about 2 min followed by gentle mixing. We placed 10 µL of cell suspension onto a microscopic slide, covered it with a glass coverslip and examined it with a fluorescence microscope using a fluorescein filter. (Higher or lower magnification may be desired depending on cell type. Nuclear morphology should be discernible). Dual staining was examined under a fluorescent microscope.

#### 2.3.6. Apoptosis Detection Using Pi Annexin V-FITC Staining of HepG2 Cells by Flow Cytometry

The day before induction of apoptosis, 1 × 10^6^ cells per well for a 6-well plate were plated using DMEM cell culture medium. After ~18 h, the wells with floating (dead) cells were removed by pipette. Then, this was replaced with a new culture medium to the original volume. The cells were treated to induce apoptosis with 160 and 320 µg/mL of sample and incubated for 24 h. Later, the cell culture medium was collected into 15 mL tubes. 

Using policeman, the cells were detached from the dish,1 mL of medium was added to each well and the contents transferred to 15 mL tubes. Then, it was centrifuged and the supernatant discarded. The cells were washed twice with cold PBS and then resuspended in 1 mL 1× binding buffer at a concentration of ~1 × 10^6^ cells/mL. We aliquoted 500 µL of cell suspension and added 10µL of PI and 5 μL Annexin V. The suspension was incubated for 15 min at room temperature in the dark. During the post-incubation, the cells were analysed by flow cytometer as soon as possible (within 1 h) [23,24,25,26,27,28,29,30].

#### 2.3.7. Gene regulation of BAX and BCL 2 in HepG2 Cell Line Treated with *W. somnifera* Extract

The concentrations of *Withania somnifera* extract and standard doxorubicin were taken as 80 µg/mL, 160 µg/mL, and 25 µM including control, respectively. 

Sample Preparation and RNA Isolation: Sample drug-treated HepG2 Cell lines were trypsinised, pelleted and supernatant was decanted. To this 0.5 mL of TRIzol was added and transferred to the tube and vortexed. Samples were allowed to stand for 5 min at room temperature. To this 0.2 mL chloroform was added per 1 mL of TRIzol used. Then, we closed the tube and vortexed it vigorously for 15 s. The tube was allowed to stand at room temperature for 5 min. We centrifuged the resulting mixture at 12,000× *g* rpm for 15 min at 4 °C. Then, we transferred the colourless upper aqueous phase to a new, clean tube.

We added an equal amount of isopropanol, mixed gently by inverting the sample 5 times and incubated it at −20 °C for 5 min. The sample was centrifuged at 12,000× *g* rpm for 15 min at 4 °C. The supernatant was discarded and the RNA pellet was washed by adding 1 mL of 70% ethanol. The sample was mixed gently by being inverted a few times, then centrifuged for 5 min at 12,000× *g* rpm at 4 °C. The supernatant was discarded by inverting the tube on clean tissue paper. Later, the pellet was left to rest for 5 min at 37 °C. The pellet was then resuspended in 25 µL of DEPC treated water.

RT-PCR: A semi-quantitative reverse transcriptase–polymerase chain reaction (RT-PCR) was carried out using the Techno Prime system to determine the levels of GAPDH, BCL II, and BAX gene mRNA expressions. 

The cDNA was synthesized from 2 µg of RNA using the Verso cDNA synthesis kit (Thermo Fischer Scientific, Waltham, MA, USA) with an oligo dT primer according to the manufacturer’s instructions. The reaction volume was set to 20 μL and cDNA synthesis was performed at 42 °C for 60 min, followed by RT inactivation at 85 °C for 5 min. Primers: (Primers synthesized at Eurofins Genomics, India). 

The PCR mixture (final volume of 20 µL) contained 1 µL of cDNA, 10 µL of Red Taq Master Mix 2x (Amplicon) and 1 µM of each complementary primer specific for BAX, BCL II and GAPDH (internal control) sequence. The samples were denatured at 94 °C for 10 min, and amplified using 40 cycles of 94 °C for 30 s, 55 °C for 30 s, and 72 °C for 30 min, followed by a final elongation at 72 °C for 10 min. The optimal numbers of cycles have been selected for amplification of these two genes experimentally so that amplifications were in the exponential range and did not reach a plateau. Ten microliters of the final amplification product were run on a 2% ethidium-stained agarose gel and photographed. Quantification of the results was accomplished by measuring the optical density of the bands, using the computerized imaging program Image J. The values were normalized to β-actin intensity levels. Table 1 given bellow mentions the sequence of the primers used.

## 3. Results and Discussion

### 3.1. Plant Extraction

The powdered plant samples were weighed to acquire 20 gm of each and were extracted in the methanolic solvent. Among all the three samples, the semi-solid extract weight of *C. Sinensis* was found to be the highest, with 1111.4 mg, and *W. somnifera* was the lowest, with 1057.6 mg for 20 g of the dry powder. *V. vinifera* weighed 1672 mg. 

### 3.2. Phytochemical Analysis

The phytochemical screening of the three plant samples revealed the presence of alkaloids, tannins, terpenoids, flavonoids, and volatility. Only for *C. Sinensis* and *V. vinifera* did volatility test positive. Phytochemicals testing positive during the analysis are known to exhibit medicinal activities on human health. The presence of flavonoids and terpenoids were previously reported for their anti-microbial, anti-mutagenic, anti-inflammatory and anti-allergic activities in various studies. Thus, the presence of these useful phytochemicals in these plants gives us a promising report (Table 2). 

### 3.3. Antioxidant Activity Using DPPH Assay

The absorbance was measured at 510 nm using a semi-autoanalyzer. The higher the IC50 value, the lower will be the relative activity in comparison to standard and vice versa. The samples of green tea and Ashwagandha leaves showed activity having an IC50 value of 14.16 µg/mL and 59.21 µg/mL, respectively. Standard quercetin showed an IC50 value of 2.866 µg/mL (Table 3). 

#### Two-Way ANOVA

A two way ANOVA was conducted between the different concentrations of samples to compare the effects of the samples on DPPH scavenging activity. There was a significant effect of different samples present at *p* < 0.05 level. 

**Figure 2:**
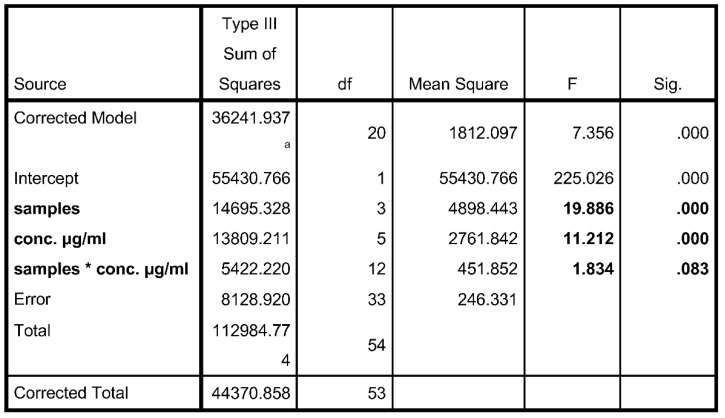
a. R Squared = 0.817 (Adjusted R Squared = 0.706).

From Figure 2, it can be said that the % inhibition of DPPH varies with different plant extracts and the concentrations used. 

Dependent variable: % inhibition.

### 3.4. α-Amylase Assay

**Figure 3:**
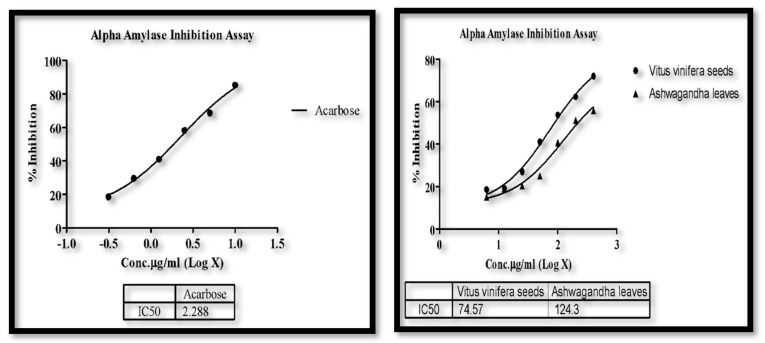
Linear graph of *V. vinifera*, *W. somnifera* and standard acarbose and plant.

The samples of *Vitus vinifera* seeds and Ashwagandha leaves showed an IC50 value of 74.57 μg/mL and 124.3 μg/mL inhibition of α-amylase, respectively. The standard acarbose has shown inhibition of α-amylase with an IC50 value of 2.288 µg/mL. Thus, *W. somnifera* shows high α-amylase activity compared to *V. vinifera*. Additionally, biochemical analysis and immunohistochemical techniques support the theory that hyperamylasaemia in is due to amylase production in carcinoma cells. Thereby we tried establishing the amylase inhibition potential of the plant extracts, which could in turn indicate their anticancer potential. [31,32]. IC50value is calculated using Graph Prism software version 5.0 by nonlinear regression analysis of % inhibition recorded for different concentrations of test substances/standard and graph plot of concentration and % inhibition was done as shown in Figure 3.

### 3.5. HPLC Analysis 

**Figure 4:**
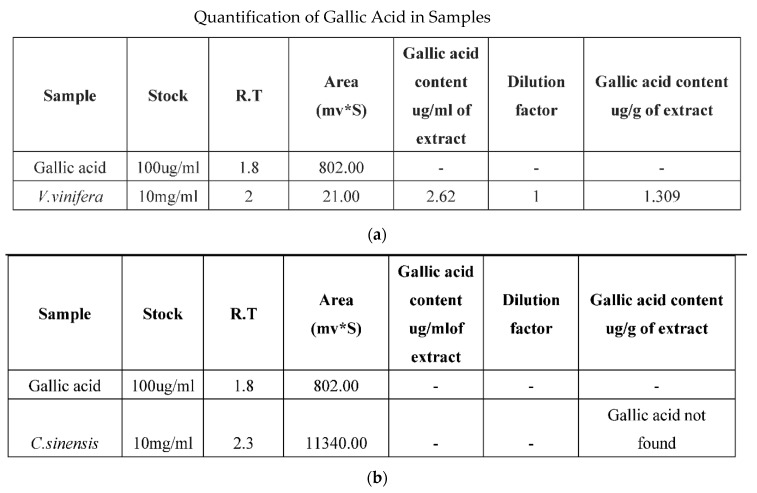

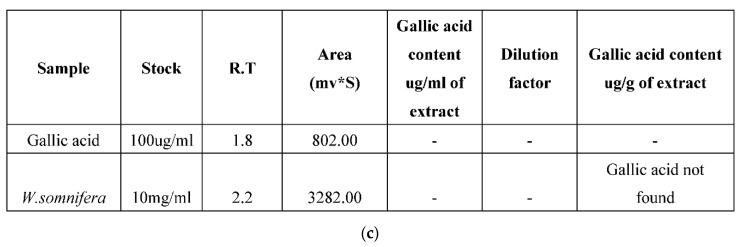
(**a**) Quantification of standard and *V. vinifera.* (**b**) Quantification of standard and *C. sinensis*. (**c**) Quantification of standard and *W. somnifera*.

**Figure 5:**
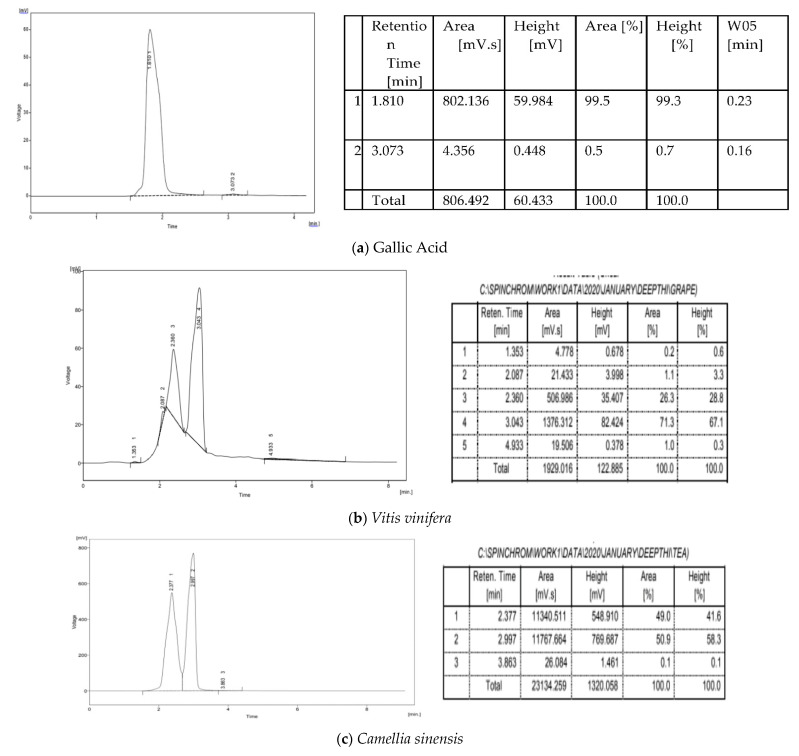

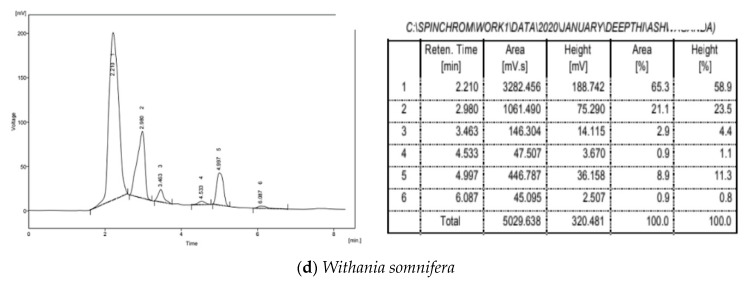
(**a**) HPLC report of gallic acid. (**b**) HPLC report of gallic acid. (**c**) HPLC report of *Camelia sinensis*. (**d**) HPLC report of *Withania somnifera.*

The HPLC report indicates the presence of the bioactive compound present in the plant sample along with gallic acid as standard reference. However, a peak corresponding to the standard is not observed in the samples. The methanolic extracts of the plant samples were subjected to HPLC analysis at 254 nm. Significant peaks were observed in all the samples, with the differences in retention time and peak suggesting that the presence of the bioactive compound was different in each sample. The retention areas of *V. vinifera*, *C. sinensis*, *W. somnifera* were during peak purity were, respectively, (2–21), (2.3–11,340), (2.2–3282). Standard gallic acid quantification was done as presented in Figure 4 [33,34,35]. The HPLC result of plant extracts has been showed in Figure 5a–d along with retention time and area covered

### 3.6. MTT Assay for HepG2 Cell Line 

The relative activity of the sample can be determined by comparing the IC50 value of the sample with a standard. The IC_50_ values for the cytotoxicity tests were derived from nonlinear regression analysis (curve fit) based on the sigmoid dose–response curve (variable) and computed using Graph Pad Prism 6 (Graph pad, San Diego, CA, USA). The Ashwagandha leaves showed an IC50 value of 153.8 µg/mL inhibition in HepG-2 cells. Standard doxorubicin showed an IC50 value of 18.8 µM inhibition in the same. *Vitus vinifera* and *C. sinensis* did not show significant activity, hence IC50 was not calculated. The morphology of the cell and its viability were observed after inducing the test concentrations of the standard and the samples, i.e., at 72 h.

**Figure 6:**
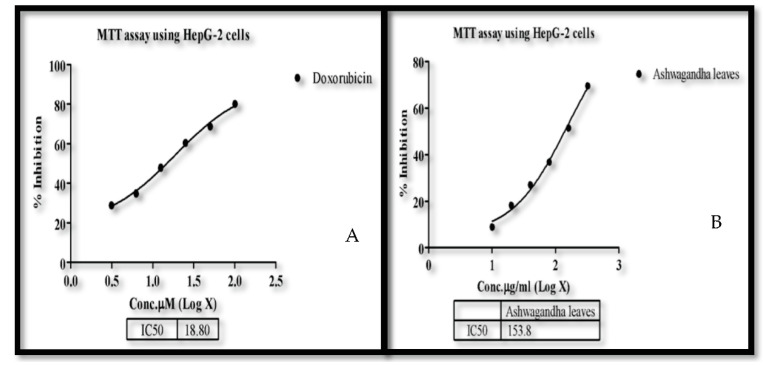
Shows the significant activity of the (**A**) standard doxorubicin and (**B**) *W. somnifera* extracts against HepG2 viable cells in the culture medium.

The observation was performed by an inverted light microscope where one can see the viable HepG2 cells in the control. Then, the cell was treated with standard doxorubicin and the three samples. Where there were shrunken cells, the initiation of the degeneration of the cell membrane and cell death or degraded cells with irregular appearance could be observed in the 10 and 320 µg/mL concentrations of different samples of treated cells. There is a comparatively greater amount of destruction or inhibition of the cell division in the media treated with *W. somnifera* samples at 10 and 320 µg/mL than the other two samples (Figure 6). Therefore, only *W. somnifera* was further taken for the cell lines studies, while the other two plant extract samples were not. (Table 4).

**Figure 7:**
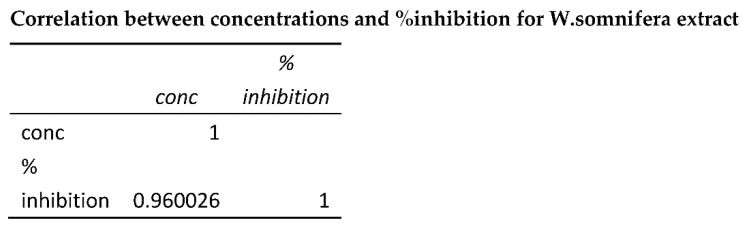
Correlation between concentration and % inhibition.

W can see that there is strong positive correlation between the concentration and % inhibition and this was calculated using Microsoft Excel 2013. (Figure 7).

### 3.7. Acridine Orange and Ethidium Bromide Dual Staining Studies Using HepG2 Cell Line 

**Figure 8:**
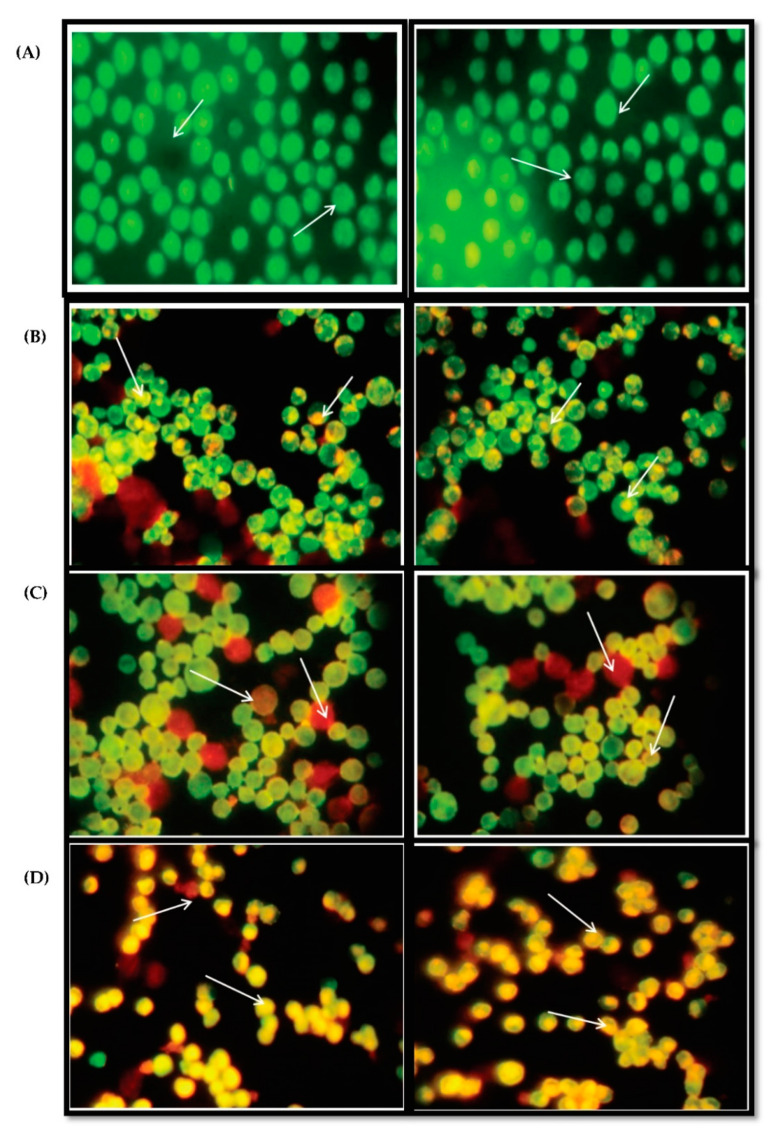
(**A**) controlHepG2 cells; (**B**) *W. Somnifera* 80 µg/mL; (**C**) *W. somnifera* 160 µg/mL; (**D**) Standard doxorubicin.

As indicated by the arrows, normal cells have the circular nucleus uniformly distributed in the centre of the cell, which is seen in the control Figure 8A. In Figure 8B showing Early stage apoptotic cells, the cell’s nucleus is showing yellow-green fluorescence by acridine orange (AO) staining and is concentrated into a crescent or granular form that is located in one side of the cells only. Staining was localized asymmetrically within the cells. In Figure 8C, it is seen that the nucleus of cells showed orange fluorescence by EtBr staining and gathered in a concentrated manner and had biased locations. This is a late apoptotic phase. The cells that have taken up complete EtBr are the necrotic cells, as in Figure 8D.

### 3.8. Apoptosis Detection Using Pi Annexin V-Fitc Staining of Hepg2 Cells by Flow Cytometry

The percentage analysis of the viable, early, late and necrotic cells as and the concentration increases were calculated, showing that the sample *W. somnifera* leaves treated at 80 µg/mL and 160 µg/mL induced early and late apoptosis in the HepG2 cells, with 8.39%, 11.33% of early apoptotic cells and 17.82%, 19.91% late apoptotic cells, respectively. Standard doxorubicin showed 52.71% late apoptotic cells in HepG2. 

**Figure 9:**
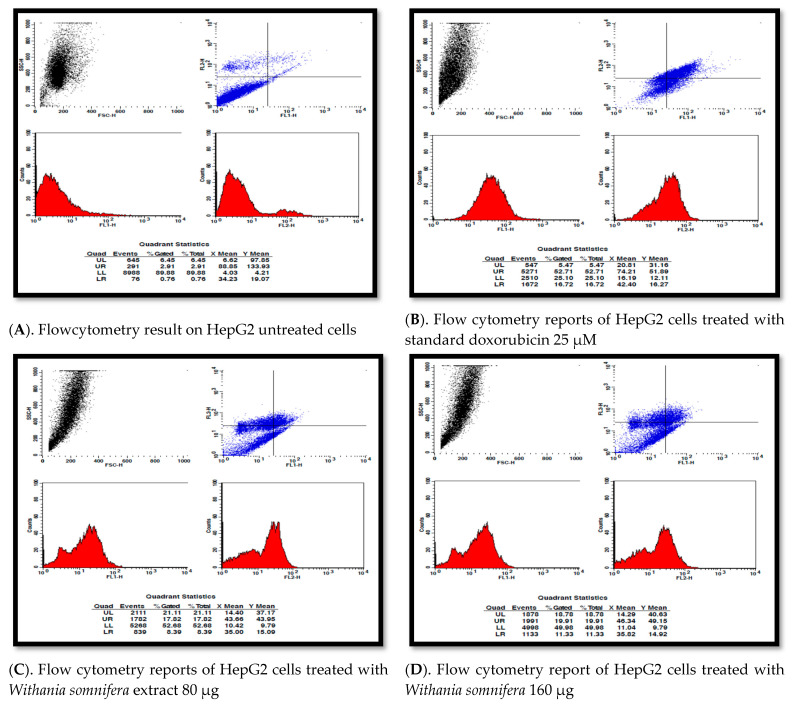
(**A**–**D**) Report of apoptosis detection by flow cytometry. FL1 is PI and FL2 is FITC axis.

**Figure 10:**
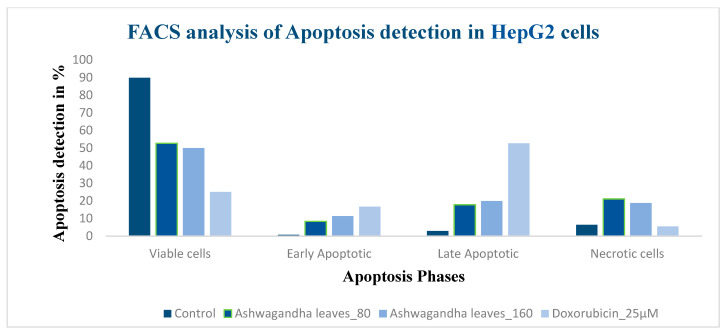
Flow cytometry analysis of apoptosis detection of HepG2 cells.

Flow cytometer was performed for the detection of apoptosis of the HepG2 cells treated with different concentrations of the samples with control. As mentioned in Figure 9A, the control exhibits the light scattered properties of cells that are untreated (SSC-H and FSC-H). The viable, early apoptotic, and late apoptotic cells are gathered in the upper left, upper right, and lower left quarters, and necrotic cells in lower right quarter, respectively, in Figure 9B–D, where Figure 9B is the standard. The cells were treated with samples at concentrations of 80 µg/mL and 160 µg/mL. (Figure 10).

### 3.9. Gene Regulation of BAX and Bcl 2 in HepG2 Cell Line Treated with Sample Ashwagandha

**Figure 11:**
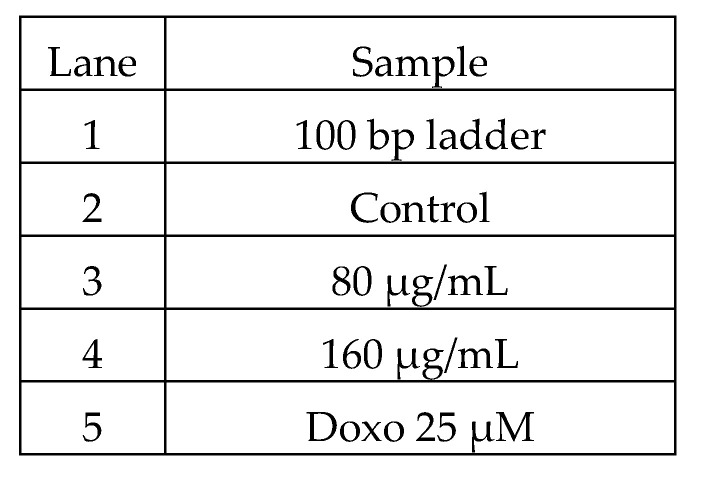
Sample loading order.

**Figure 12:**
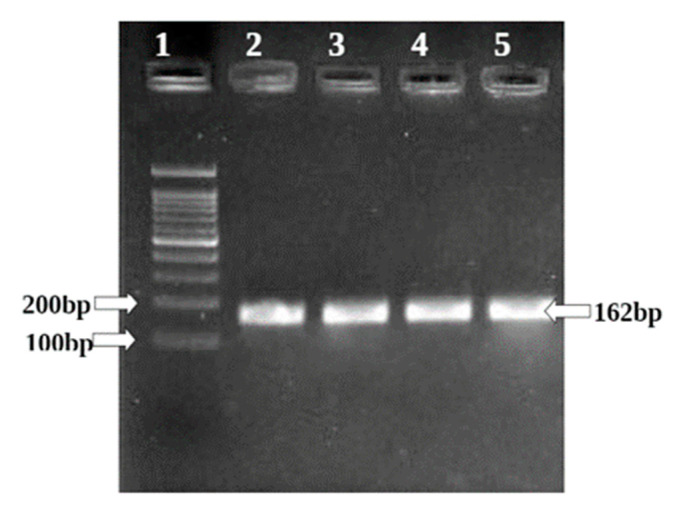
Amplification of GAPDH in HepG2 cell line.

**Figure 13:**
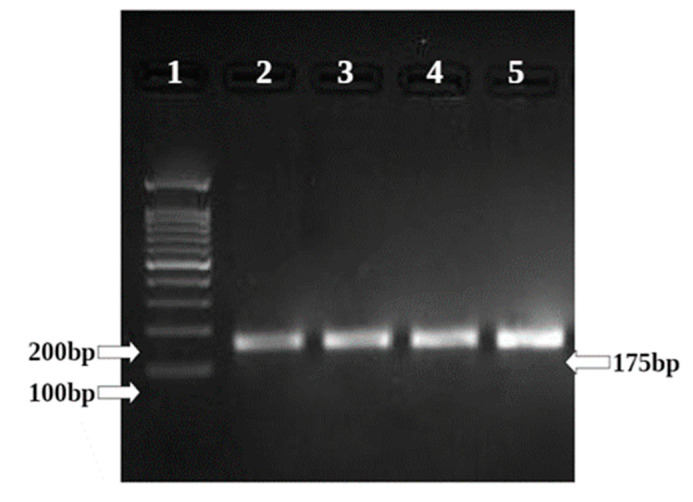
Gene regulation expression of BAX in HepG2 cell line.

**Figure 14:**
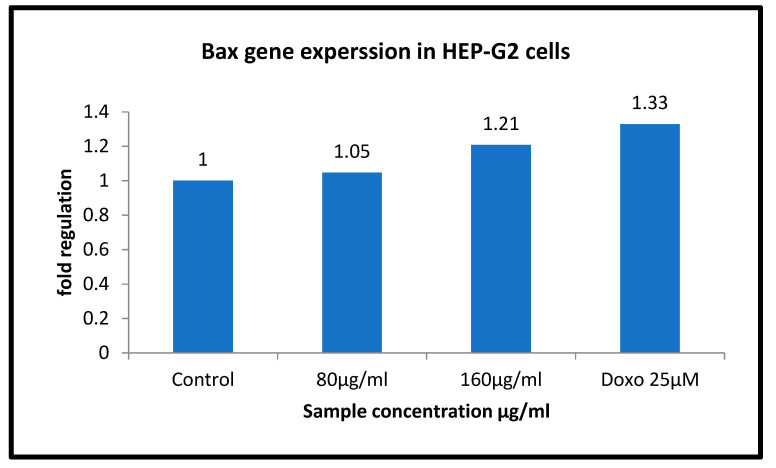
Graphical representation of BAX gene expression.

BAX and BCL II gene expression is quantified in the HepG2 cell line by semi-quantitative PCR. (Figure 11 and Figure 12) The internal control GAPDH gene was used to normalize the BAX and BCL II gene expression levels. The *W. somnifera* treatment at different concentrations on the HepG2 cell line has shown significant regulation of the BAX gene. At 80 µg/mL and 160 µg/mL concentrations, the BAX gene expression has shown upregulation to 1.05 and 1.21, respectively, in the HepG2 cell line (Figure 13 and Figure 14). 

**Figure 15:**
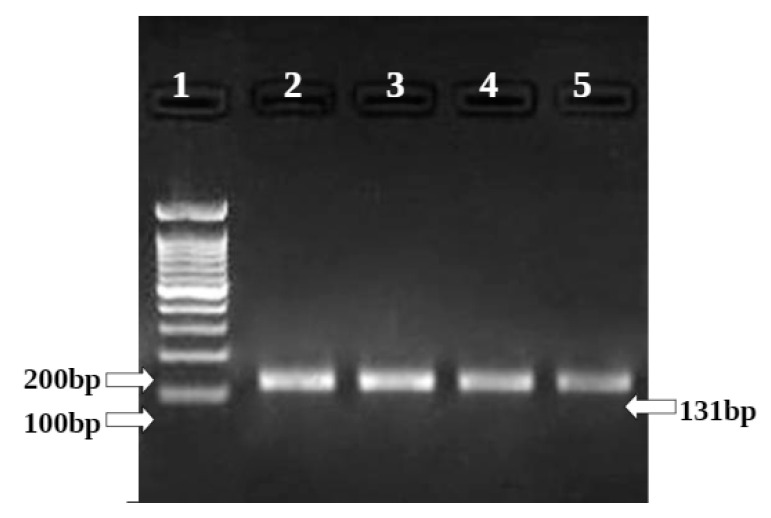
Gene regulation expression of Bcl2 gene in HepG2 cell line.

**Figure 16:**
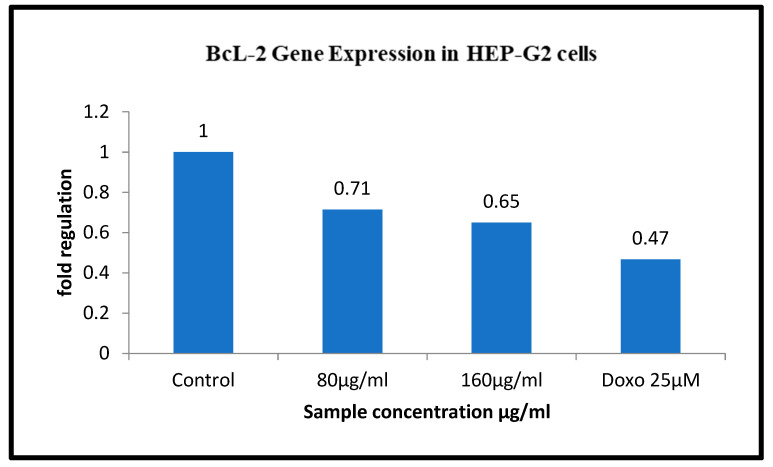
Graphical representation of BCl2 gene expression.

The Ashwagandha treatment at different concentrations on the HepG2 cell line has shown significant downregulation of the BCL II gene. Concentrations of 80 µg/mL and 160µg/mL showed downregulation of BCl2 gene expression to 0.71 and 0.65 in the HepG2 cell line, respectively. The positive control doxorubicin treatment at 25 µM on the HepG2 cell line showed significant upregulation in BAX genes (to 1.33) and downregulation in BCL II genes (to 0.47) in the HepG2 cell line (Table 5 and Table 6) and (Figure 15 and Figure 16).

This study was carried to evaluate the presence of anti-carcinogenic components in the plant extract of *Camellia sinensis*, *Withania somnifera* leaves, and *Vitis vinifera* seeds. These plants we chosen for the study due to early reports which showed the presence of anti-cancerous properties since they are rich in antioxidant phytoconstituents.

All the plant samples for the study were extracted in the methanolic solvent and yield was noted. Then, they were subjected to the phytochemical screening, which indicated the presence of active compounds such as tannins, alkaloids, terpenoids, and flavonoids. Later, *Camellia sinensis* and *Withania somnifera* revealed very good antioxidant activity in the DPPH assay. While *Vitis vinifera* and *W. somnifera* show α-amylase inhibition, the IC50 value of *C. sinensis* was not calculated due to its lower % inhibition.

Then, the extracts were examined for the presence of bioactive compounds in the three plant extracts corresponding to gallic acid as reference compound. The methanolic extracts of the plant samples were subjected to the HPLC analysis at 254 nm. Significant peaks were observed in all the samples, with the differing retention times and peaks suggesting the presence of the different bioactive compounds in each sample. This HPLC result suggested the presence of compounds responsible for anti-carcinogenic activity. Then, the MTT assay which was conducted for all the three plant extracts, in which, *Withania somnifera* extract exhibited higher activity than the other extracts. Hence, *W. somnifera*, was taken further for study on HepG2 cell line. The morphological observations made during the assay revealed that there is a significant decrease in the viability of the cell as the concentration of the induced *W. somnifera* extract increases, thus indicating the anti-proliferation activity against the HepG2 cell line. The treatment of the HepG2 cell line with the *W. somnifera* extract was performed and FACS analysis was carried out to detect the apoptotic pathway of the cell line induced with the plant extract at different concentrations, exhibiting promising results. The observation conducted during the MTT assays of different concentrations showed that *W. somnifera* extract has anti-proliferative activity against the HepG2 cancer cell line. The later the expression of BAX (pro-apoptotic) and BCl2 (anti-apoptotic) genes treated with *W. somnifera* also showed significant upregulation of the BAX and downregulation of the BCL2 genes along with the standard doxorubicin. The pro-apoptotic BAX gene showed increased gene expression following treatment with *W. somnifera*, while BCl2, the anti-apoptotic gene, was downregulated, indicating that this plant extract induced inhibition of the proliferation of the cancer cells. 

*W. somnifera*, being one of the important medicinal herbs in India, also has other biological activities such as anti-aging, anti-inflammatory and anti-fatigue properties. The result obtained in this study shows the presence of the bioactive compounds which are effective in the prevention of the proliferation of HepG2 cells in vitro. It acts as detoxifying agent in eliminating the toxins responsible for the initiation of cancer cells. *W. somnifera* has expressed positive results in encountering other types of cancer such as breast cancer, lung cancer, and renal cancer. The development of cancer cells is multifactorial and occurs due to the causative agents released during stress, exposure to radiation or infection. This study hypothesizes that *W. somnifera* can be expressed as a potential agent for the elimination of the excess oxidative species and toxins responsible for cancer initiation. 

The study shows that the extract of *W. somnifera* leaves exhibits significant activity as an anti-carcinogenic agent. This might be further studied for the identification and isolation of the bioactive component responsible for its antioxidant activity. This might pave the way further for enhancing this particular bioactive compound for the treatment of cancer.

## 4. Conclusions

In conclusion, among the three plant extracts, *W. somnifera* leaves extract exhibited the highest carcinogenic cell inhibition via apoptosis when induced to the HepG2 cell line in vitro, while the *C. sinensis* and *V. vinifera* extracts showed lesser inhibition activity in DPPH and MTT assay, hence, were not further subjected to cell line study. However, methanolic extract of *W. somnifera*, which is rich in phyto-constituents, has shown enhancing apoptosis of the HepG2 cell line. As a result, there is upregulation of BAX genes, inducing apoptosis, and downregulation of BCl2 gene in the HepG2 cell line. This study exhibited the inhibition of the HepG2 cell proliferation due to the upregulation of the BAX gene and decreased BCl2 gene expression. Therefore, *W. somnifera* has proven its potential anticancer properties in vitro for HepG2 cells and also for various types of cancers in other studies. Further investigation of the mechanistic basis of the above observed activities in combination with in vivo studies may be carried out to gain a deeper insight into these bioactive compounds and aid is an affordable line of drug development for hepatocellular carcinoma. 

## Figures and Tables

**Table 1:** Details of primer with the sequence and product size.

Gene	Primer Pair	Sequence	Tm	Product Size (bp)
GAPDH	FP	GTCCAGTTAATTTCTGACCT	47.7	162 bp
	RP	GCTTTGTACATGGTATTCAC	47.7	
BCL II	FP	CTGGTGGACAACATCGCTCTG	61.4	131 bp
	RP	GGTCTGCTCACCTCACTTGTG	61.4	
BAX	FP	GGCACCTGAGCTGACCTTGG	47.7	175 bp
	RP	GAGGAAGTCCAGTGTCCAGC	47.7	

**Table 2:** Phytochemical screening of three plant extracts for bioactive compound.

Types of Tests	*Green tea*	*Vitus vinefera*	*Ashwagandha*
Alkaloids	+	+	+
Carbohydrates	-	-	-
Tannins	+	+	+
Terpenoids	+	+	+
Glycosides	-	-	-
Steroids	-	-	-
Saponins	-	-	-
Flavonoids	+	+	+
Cardiac glycosides	-	-	-
Mucilage test	-	-	-
Volatile oil	+	+	-

Note: +: Positive, -: Negative.

**Table 3:** Antioxidant activity using DPPH assay.

Sample	Conc. (μg/mL)	OD @ 510 nm	% Inhibition	IC50 (µg/mL)
**Control**	0	0.912	0.00	2.866
**Quercetin**	0.3125	0.521	42.87	
	0.625	0.430	52.85	
	1.25	0.391	57.13	
	2.5	0.324	64.47	
	5	0.224	75.44	
	10	0.145	84.09	
***Vitus vinifera*** **seeds**	3.125	0.845	7.35	-
	6.25	0.746	18.20	
	12.5	0.684	25.00	
	25	0.568	37.72	
	50	0.532	41.67	
	100	0.488	46.49	
***Camelia sinensis*** **leaves**	3.125	0.806	11.62	14.16
	6.25	0.698	23.46	
	12.5	0.497	45.50	
	25	0.361	60.42	
	50	0.231	74.67	
	100	0.104	88.60	
***Withania somnifera*** **leaves**	3.125	0.857	6.03	59.21
	6.25	0.730	19.96	
	12.5	0.702	23.03	
	25	0.648	28.95	
	50	0.548	39.91	
	100	0.397	56.47	

Note: % inhibition and IC50 value of plant extracts and quercetin as standard.

**Table 4:** MTT assay report.

HepG-2	Standard			
Compound Name	Conc. µM	OD @ 590 nm	% Inhibition	IC50 in µM
**Control**	0	0.9731	0.00	18.8
***Doxorubicin***	3.125	0.692	28.88	
	6.25	0.634	34.86	
	12.5	0.507	47.88	
	25	0.385	60.46	
	50	0.305	68.69	
	100	0.194	80.02	
**HepG-2**				
**Compound Name**	**Conc. µg/ml**	**OD @ 590 nm**	**% Inhibition**	**IC50 µg/mL**
**Control**	0	0.973	0.00	IC50 was not calculated to lesser % inhibition
***Vitis vinifera***	10	0.900	7.50	
	20	0.865	11.03	
	40	0.753	22.58	
	80	0.720	26.01	
	160	0.667	31.38	
	320	0.577	40.62	
***Camelia sinensis***	10	0.906	6.82	IC50 was not calculated to lesser % inhibition
	20	0.848	12.76	
	40	0.776	20.19	
	80	0.684	29.68	
	160	0.642	34.00	
	320	0.610	37.29	
***Ashwagandha leaves***	10	0.886	8.88	153.80
	20	0.796	18.20	
	40	0.710	27.00	
	80	0.615	36.76	
	160	0.475	51.34	
	320	0.296	69.51	

**Table 5:** Relative expression of BAX gene treated with Ashwaganda in HepG2.

HepG2				
Sample Conc. µg/mL	Band Intensity of PCR Amplicons		Normalised	Relative Gene Expression
	GAPDH	BAX		
Control	12,631.08	17,305.61	1.370	1
80 µg/mL	15,367.00	22,031.13	1.434	1.05
160 µg/mL	14,598.85	24,165.42	1.655	1.21
Doxo 25 µM	16,118.44	29,342.88	1.820	1.33

**Table 6:** Relative expression of BCL II gene treated with Ashwagandha in HepG2.

HepG2				
Sample Conc. µg/mL	Band Intensity of PCR Amplicons		Normalised	Relative Gene Expression
	GAPDH	BCl2		
Control	12,631.08	18,823.78	1.490	1
80 µg/mL	15,367.00	16,342.77	1.063	0.71
160 µg/mL	14,598.85	14,123.93	0.967	0.65
Doxo 25 µM	16118.44	11209.12	0.695	0.47

## Data Availability

Not available.
